# Prediction of Response to Treatment by Gene Expression Profiling of Peripheral Blood in Patients with Microscopic Polyangiitis

**DOI:** 10.1371/journal.pone.0063182

**Published:** 2013-05-17

**Authors:** Akihiro Ishizu, Utano Tomaru, Taichi Murai, Tomohiro Yamamoto, Tatsuya Atsumi, Takashi Yoshiki, Wako Yumura, Kunihiro Yamagata, Hidehiro Yamada, Shunichi Kumagai, Manae S. Kurokawa, Machi Suka, Hirofumi Makino, Shoichi Ozaki, for JMAAV

**Affiliations:** 1 Faculty of Health Sciences, Hokkaido University, Sapporo, Japan; 2 Department of Pathology, Hokkaido University Graduate School of Medicine, Sapporo, Japan; 3 GeneticLab Co., Ltd., Sapporo, Japan; 4 Department of Internal Medicine II, Hokkaido University Graduate School of Medicine, Sapporo, Japan; 5 Department of Nephrology, International University of Health and Welfare Hospital, Tochigi, Japan; 6 Department of Nephrology, Graduate School of Comprehensive Human Sciences, University of Tsukuba, Tsukuba, Japan; 7 Division of Rheumatology and Allergology, Department of Internal Medicine, St. Marianna University School of Medicine, Kawasaki, Japan; 8 Department of Clinical Pathology and Immunology, Kobe University Graduate School of Medicine, Kobe, Japan; 9 Clinical Proteomics and Molecular Medicine, St. Marianna University Graduate School of Medicine, Kawasaki, Japan; 10 Department of Public Health and Environmental Medicine, The Jikei University School of Medicine, Tokyo, Japan; 11 Department of Medicine and Clinical Science, Okayama University Graduate School of Medicine Dentistry and Pharmaceutical Sciences, Okayama, Japan; Keio University School of Medicine, Japan

## Abstract

The JMAAV study was an open-labeled prospective clinical trial, which proposed severity-based treatment protocols for patients with microscopic polyangiitis (MPA). The results suggest that the proposed protocols are useful (remission rate: 89.4%), but are also indicative of relapse or patient demise regardless of the treatment (recurrence rate: 19.0%; mortality rate: 10.6%). The aim of this study is to develop the method to predict response to the treatment in patients with MPA. In the present study, transcriptome analysis was performed using peripheral blood from patients enrolled in the JMAAV study before and 1-week after the beginning of treatment. The gene expression profile before treatment was not directly related to the response to the treatment. However, when the samples from 9 patients with good response (persistent remission for 18 months) were examined, the expression of 88 genes was significantly altered by the treatment. Thirty statistically reliable genes were selected, and then the alteration of expression by the treatment was examined among 22 patients, including 17 with good response, which was defined as persistent remission for 18 months and 5 with poor response, which was defined as relapse after remission or no remission. Discrimination analysis between the alteration of expression of the 30 genes by the treatment and the response identified a combination of 16 genes as the most valuable gene set to predict the response to the treatment. This preliminary study identified IRF7, IFIT1, IFIT5, OASL, CLC, GBP-1, PSMB9, HERC5, CCR1, CD36, MS4A4A, BIRC4BP, PLSCR1, DEFA1/DEFA3, DEFA4, and COL9A2 as the important genes that can predict the response to the treatment in patients with MPA at an early point during the therapy.

## Introduction

The spectrum of anti-neutrophil cytoplasmic autoantibody (ANCA)-associated vasculitis (AAV) includes microscopic polyangiitis (MPA), eosinophilic granulomatosis with polyangiitis (EGPA, Churg-Strauss syndrome), and granulomatosis with polyangiitis (GPA, Wegener's granulomatosis) [1]. The two major antigens of ANCA are myeloperoxidase (MPO) [2] and proteinase 3 (PR3) [3]. MPO-ANCA is often detected in the sera of patients with MPA and EGPA; while, PR3-ANCA is a useful marker for GPA. Although it remains unsolved why ANCA is produced, immunological mechanisms are considered to be involved in the development of AAV. Therefore, corticosteroids and immunosuppressive agents have been used as treatments for AAV. Based on previous clinical trials, the standard protocol of treatment for AAV was established in Western countries [4]–[6].

The prevalence of MPA is strikingly higher in Japanese population compared to the Caucasoid [7]. Accordingly, clinical trials to establish a guideline for the management of patients with this subtype of AAV should be held in Japan. Ozaki and colleagues instituted a Japanese study group for MPA and conducted an open-labeled prospective clinical trial, the JMAAV study (The University Hospital Medical Information Network, Clinical Trials Registry; http://www.umin.ac.jp/ctr/index-j.htm, registration number ID 000000867) [8]. In the JMAAV study, patients newly diagnosed with MPA were stratified into 3 categories based on disease severity, including mild form, severe form, and most severe form. The mild form included patients with slight disorder of one or more organs, renal-limited type (except for rapidly progressive glomerulonephritis (RPGN)), and pulmonary-limited type (except for pulmonary hemorrhage). The severe form included patients with generalized type (MPA with involvement of more than 2 organs), pulmo-renal type (glomerulonephritis plus either limited pulmonary hemorrhage or extended interstitial pneumonia), and RPGN type. The most severe form included patients with diffuse alveolar hemorrhage, intestinal perforation, acute pancreatitis, cerebral hemorrhage, or concurrent presence of anti-glomerular basement membrane antibodies. This form also included patients with the severe form who were resistant to the severity-based treatment protocol described below.

After the establishment of diagnosis, the patients were treated according to the following protocols. 1) Mild form: Low-dose corticosteroids (0.3–0.6 mg/kg/day) were administered. Oral immunosuppressive agents (cyclophosphamide or azathioprine, 0.5–1.0 mg/kg/day or 25–75 mg/day, respectively) were optional. 2) Severe form: High-dose corticosteroids (0.6–1.0 mg/kg/day) and oral cyclophosphamide (0.5–2.0 mg/kg/day) were given. Intravenous methylprednisolone (0.5–1.0 g/day for 3 days) was considered as an alternative. Instead of oral administration, the use of intravenous cyclophosphamide (0.5–0.75 g/m^2^ monthly) was also allowed. 3) Most severe form: Plasmapheresis (2.0–3.0 L/day for 3 days) was employed together with the regimen for the severe form described above.

Fifty-two patients were registered to the JMAAV study, but 4 were excluded due to the exclusive prescriptions. The remaining 48 patients were divided into the mild form (n = 23), severe form (n = 23), and most severe form (n = 2) groups. Treatment was administered according to the stated protocol. They were followed-up for 18 months. Since 1 patient in the mild form was lost to follow-up within 6 weeks, the study population for further analysis consisted of the remaining 47 patients. Remission, which was defined as the absence of clinical manifestations of active vasculitis (Birmingham Vasculitis Activity Score 2003: 0 or 1 point), was achieved in 42 out of 47 patients (remission rate: 89.4%). Among the 42 patients, 8 patients showed relapse of the disease (recurrence rate: 19.0%). Relapse was defined as the recurrence or development of at least one manifestation of vasculitis. The involvement of each organ was diagnosed as described elsewhere [8]. Ultimately, 5 of the 47 patients died (mortality rate: 10.6%).

These results suggest that the proposed severity-based protocols are applicable for patients with MPA, but the possibility of relapse is indicated and, in the worst scenario, death may occur regardless of the treatment. We considered that if the response to the treatment would be predicted prior to the beginning of treatment or at an early point during the therapy, careful follow-up or application of additional regimens to the treatment could expectedly improve the outcome.

## Materials and Methods

### Ethics statement

Peripheral blood was obtained from patients with written informed consent in accordance with the Declaration of Helsinki. The use of human materials was permitted by the Institutional Clinical Research Committee in Hokkaido University Hospital (No. 0903-0398).

### Patient cohorts and blood samples

The list of patients registered to the JMAAV study is shown in Table 1. Peripheral blood samples (10 mL) were obtained from 39 out of the 47 patients with MPA before and 1-week after the beginning of treatment. Total RNAs were extracted using PAXgene Blood RNA System (BD, Franklin Lakes, NJ). Among the 39 pairs of blood samples, 5 pairs did not suit for the following assay because of poor quality or low amount of the extracted RNAs. The 34 patients with paired RNA samples were randomly divided into 2 cohorts, namely Cohort 1 and Cohort 2. The comparison of clinical characteristics between the 2 cohorts, including age, sex, and serum levels of creatinine and MPO-ANCA, is summarized in Table 2. Although the gender distribution seemed to be imbalance between the 2 cohorts, there was no statistically significant difference (p = 0.0543 in Fisher's exact test). In addition, age and serum levels of creatinine and MPO-ANCA were equivalent between the 2 cohorts (p = 0.3077, p = 0.5055, and p = 0.7026, respectively, in Mann-Whitney *U*-test). Since there was no significant gender difference in age and serum levels of creatinine and MPO-ANCA between male and female genders in Cohort 1 and Cohort 2 (Table S1), the imbalance of gender between the 2 cohorts, if any, was not likely to be a problem.

**Table 1:** Patients registered to JMAAV study.

Case No.*	Age/Sex	Disease form (Involved organs**)	Weeks for remission	Relapse	Time of relapse (months)	Response	Cohort
1	72/M	severe (L/K/N)	6	−		good	
2	75/F	severe (L/K)	6	−		good	1
3	64/M	excluded					
4	76/F	severe (E/L/K/N)	6	−		good	
5	73/F	mild (N)	6	−		good	1
6	no record	mild (M)	6	−		good	
7	no record	severe (K/S)	6	−		good	1
8	62/F	mild (M)	not achieved			poor (dead^1^)	1
9	57/F	mild (L)	6	−		good	1
10	84/F	excluded					
11	72/F	mild (L/M)	6	+	9	poor	
12	73/M	mild (L/K/S)	6	−		good	2
13	77/F	mild (B/N)	24	+	9	poor	1
14	62/F	severe (K)	6	−		good	1
15	74/F	severe (L/K)	6	+	6	poor	1
16	57/M	severe (L/K/N)	6	+	6	poor	2
17	78/M	mild (K)	6	−		good	2
18	70/F	severe (L/K/N)	6	+	6	poor (dead^2^)	2
19	51/F	severe (K)	6	−		good	2
20	60/F	mild (L)	6	−		good	2
21	71/F	most severe (B/L/K/I)	not achieved			poor (dead^3^)	2
22	68/M	severe (K)	6	−		good	2
23	75/F	mild (K/N/J)	6	−		good	2
24	76/F	mild (K/N)	6	−		good	2
25	72/M	severe (L/K/N)	12	−		good	2
26	67/M	mild (N/S/M)	6	−		good	2
27	70/M	mild (L/M)	6	−		good	2
28	45/F	mild (K)	dropped				
29	76/M	severe (K)	6	−		good	
30	71/M	severe (K)	6	−		good (dead^4^)	
31	69/F	mild (K/N)	6	−		good	2
32	72/F	severe (L/K/N)	6			dead^5^	
33	64/F	excluded					
34	62/M	severe (E/L/K)	6	−		good	2
35	58/F	mild (L/H)	6	−		good	
36	79/F	mild (L/K)	6	−		good	2
37	58/F	severe (L/K)	12	−		good	2
38	63/F	severe (K/N/M/Li)	6	−		good	2
39	71/F	severe (K)	not achieved	dropped			
40	56/M	severe (K)	not achieved			poor^6^	
41	70/M	severe (K/N/S)	12	−		good	2
42	68/M	severe (K)	12	−		good	2
43	74/M	severe (L/K/N)	12	dropped			
44	80/F	excluded					
45	75/F	mild (L/N/M)	6	+	9	poor	2
46	64/F	mild (L)	12	+	7	poor	2
47	26/F	mild (B/K/N/S/M/J)	6	−		good	1
48	62/M	severe (L/K/N/M)	6	−		good	1
49	55/F	mild (E/L/N/S)	6	−		good	1
50	62/F	most severe (L/K/N)	6	−		good	1
51	58/F	mild (L/K/J)	6	+	6	poor	
52	65/M	mild (K/N)	6	−		good	

Patients died by.

1 Interstitial pneumonia at 3 months,

2 opportunistic infection at 11 months,

3 cerebral bleeding at 9 days,

4 respiratory failure without relapse at 10 months, and

5 cerebral bleeding due to atherosclerosis at 10 weeks.

6 Persistent hemodialysis was introduced to this patient from 1 week after diagnosis.

* The case numbers are different from the patient numbers in Takakuwa *et al.* paper (Takakuwa *et al.* Arthritis Rheum 63:3613–3624, 2011).

** Letters represent organs as follows; B: brain, E: eye/ear/nose, L: lung, K: kidney, N: peripheral nervous system, S: skin, M: muscle, I: intestine, J: joints, and Li: liver/gallbladder/pancreas.

**Table 2:** Comparison of clinical characteristics between cohorts.

Clinical characteristics	Cohort 1	Cohort 2	p-value
Age	62.3±14.2	68.0±7.2	0.3077*
Sex (M/F/no record)	1/10/1	10/12/0	0.0543**
Serum creatinine (mg/dL)	1.73±1.79	1.84±1.38	0.5055*
Serum MPO-ANCA (U/mL)	334.9±291.2	293.5±211.7	0.7026*

* Mann-Whitney *U*-test.

** Fisher's exact test.

#### Cohort 1

The blood samples from this cohort were subjected to gene chip analysis in order to discover genes relevant to the response to the treatment. This cohort included 6 patients categorized into the mild form, 5 patients into the severe form, and 1 patient into the most severe form. Remission was achieved in 11 patients, but the disease relapsed in 2 of them. The time of relapse is shown in Table 1. Remission was not achieved in 1 patient. In the present study, persistent remission for 18 months was regarded as good response. On the other hand, relapse or no induction of remission was regarded as poor response. Accordingly, this cohort included 9 patients with good response and 3 patients with poor response.

#### Cohort 2

The blood samples from this cohort were subjected to quantitative expression analysis concerning the genes listed in Cohort 1. For this purpose, low density array technology was applied. Subsequently, data mining was performed to identify the most valuable genes to predict the response to the treatment. This cohort included 11 patients categorized into the mild form, 10 patients into the severe form, and 1 patient into the most severe form. Remission was achieved in 21 patients, but the disease relapsed in 4 of them. Remission was not achieved in 1 patient. Accordingly, this cohort included 17 patients with good response and 5 patients with poor response.

### Gene chip analysis

GeneChip Human Genome Focus Array (Affymetrix, Santa Clara, CA) was used. This gene chip was equipped with 8,793 genes related to inflammation and immune response [9]. Raw data of all samples were imported into GeneChip Operating Software (Affymetrix). Each signal value was pre-normalized by MAS5.0 method, which is the manufacturer's recommended method for pre-normalization of the array data. After the pre-normalization, the array data were imported into GeneSpring GX7.3.1 Software (Agilent Technologies, Santa Clara, CA). Signal values less than 0.01 were regarded as 0.01 because minus signals are nonsense in biology. In this procedure, 0.01 was employed instead of 0 toward operation in which the corrected values would be used as denominators. For normalization per chip, each signal value was divided by the 50^th^ percentile value in the chip. For the specific detection of each gene, the gene chip was equipped with 11 to 20 probe pairs per gene, including perfect match (PM) probes and mismatch (MM) probes. The sequence of the paired PM and MM probes was identical, except for a change to the Watson-Crick complement in the middle of the MM probe sequence. For normalization per gene, signal values of PM probes were divided by the median value of the signal of MM probes.

### Low density array analysis

The real-time RT-PCR-based TaqMan Low Density Array (Applied Biosystems, Carlsbad, CA) was applied to quantify the expression of 30 genes listed by the gene chip analysis (Table 3). The accession numbers in Table 3 belonged to GenBank repository. The low density array data were analyzed as follows. First, the expression level of the target gene was standardized by the expression level of the house-keeping β-actin gene. For this purpose, the Ct value of real-time PCR was applied. The Ct value represents the cycle number in which the PCR products reach the threshold level [10]. The expression level of the target gene was shown as ΔCt (ΔCt = Ct value of the target gene – Ct value of the β-actin gene). Next, the changed amount of expression of the target gene by the treatment was shown as ΔΔCt (ΔΔCt = ΔCt 1-week after the beginning of treatment – ΔCt before treatment). It is considered that when ΔΔCt is 1, the expression level of the target gene before treatment is 2-fold higher than 1-week after the beginning of treatment. Accordingly, when the expression level of the target gene before treatment is set as 1, the fold expression of the target gene 1-week after the beginning of treatment is shown as 2^−ΔΔCt^. Subsequently, the outcome was replaced by a dummy number; wherein, “good outcome (persistent remission)” was regarded as 0 and “poor outcome (relapse after remission or no remission)” as 1.

**Table 3:** List of 30 genes equipped with low density array.

Alteration	p-Value	Gene symbol	Description	Accession No.*
Decrease	0.00441	CLC	Charcot-Leyden crystal protein	NM_001828
Decrease	0.00692	GBP1	Guanylate binding protein 1, interferon-inducible, 67 kDa	NM_002053
Decrease	0.00692	NGFRAP1	Nerve growth factor receptor (TNFRSF16) associated protein 1	NM_206917 NM_206915 NM_014380
Decrease	0.00692	IFIT1	Interferon-induced protein with tetratricopeptide repeats 1	NM_001548
Decrease	0.00696	PSMB9	Proteasome (prosome, macropain) subunit, beta type, 9 (large multifunctional peptidase 2)	NM_002800
Decrease	0.0106	CCR3	Chemokine (C-C motif) receptor 3	NM_001837 NM_178329
Decrease	0.0149	TNFSF10	Tumor necrosis factor (ligand) superfamily, member 10	NM_003810
Decrease	0.0149	MX1	Myxovirus (influenza virus) resistance 1, interferon-inducible protein p78 (mouse)	NM_002462
Decrease	0.0149	HERC5	Hect domain and RLD 5	NM_016323
Decrease	0.0160	IFIT3	Interferon-induced protein with tetratricopeptide repeats 3	NM_001549NM_001031683
Decrease	0.0200	IRF7	Interferon regulatory factor 7	NM_001572 NM_004029 NM_004031
Decrease	0.0200	OAS1	2′,5′-oligoadenylate synthetase 1, 40/46 kDa	NM_016816 NM_002534 NM_001032409
Decrease	0.0206	CCR1	Chemokine (C-C motif) receptor 1	NM_001295
Decrease	0.0234	CD36	CD36 antigen (collagen type I receptor, thrombospondin receptor)	NM_001001548 NM_001001547 NM_000072
Decrease	0.0234	MS4A4A	Membrane-spanning 4-domains, subfamily A, member 4	NM_024021 NM_148975
Decrease	0.0234	IFIH1	Interferon induced with helicase C domain 1	NM_022168
Decrease	0.0234	IFIT5	Interferon-induced protein with tetratricopeptide repeats 5	NM_012420
Decrease	0.0234	EMR1	Egf-like module containing, mucin-like, hormone receptor-like 1	NM_001974
Decrease	0.0278	OAS2	2′-5′-oligoadenylate synthetase 2, 69/71 kDa	NM_016817 NM_002535 NM_001032731
Decrease	0.0278	BIRC4BP	XIAP associated factor-1	NM_017523 NM_199139
Decrease	0.0284	OAS3	2′-5′-oligoadenylate synthetase 3, 100 kDa	NM_006187
Decrease	0.0303	MMD	Monocyte to macrophage differentiation-associated	NM_012329
Decrease	0.0324	HIST1H3H	Histone 1, H3h	NM_003536
Decrease	0.0324	PLSCR1	Phospholipid scramblase 1	NM_021105
Decrease	0.0324	MT2A	Metallothionein 2A	NM_005953
Decrease	0.0418	OASL	2′-5′-oligoadenylate synthetase-like	NM_003733 NM_198213
Increase	0.0234	COL9A2	Collagen, type IX, alpha 2	NM_001852
Increase	0.0324	DEFA4	Defensin, alpha 4, corticostatin	NM_001925
Increase	0.0324	VSIG4	V-set and immunoglobulin domain containing 4	NM_007268
Increase	0.0490	DEFA1DEFA3	Defensin, alpha 1Defensin, alpha 3, neutrophil-specific	NM_004084 NM_005217

* GenBank.

After these preparations, discrimination analysis was conducted concerning 22 patients in Cohort 2 using the fold expression of 16 genes randomly extracted from the 30 genes. In this analysis, the influence of the target gene on the prediction of the response to the treatment was calculated. Thereafter, the gene which showed the minimum influence on the prediction was replaced by another gene in the remaining 14 genes. This operation was repeated until all genes were used. Up to this, 15 combinations of genes consisting of 16 genes were generated. Subsequently, the gene with the minimum influence on the prediction was excluded one by one until the last gene remained, which resulted in the generation of another 15 combinations of genes consisting of diverse number of genes (15-1). In total, 30 combinations of genes (model #1 – model #30) were generated, and then were examined for prediction of the response to the treatment. In order to identify the most adequate model among the 30 combinations, Akaike Information Criterion (AIC) was applied. AIC is one of the suitable indices to evaluate such a model, and the algorithm showing the smallest AIC value is considered to be the most desirable [11], [12].

Lastly, in order to identify the best predictors of the response to the treatment among the genes listed in Table 3, logistic analysis was conducted concerning 22 patients in Cohort 2, including 17 with good response and 5 with poor response. Discrimination analysis and logistic analysis were performed using the add-in Excel software 2012 (SSRI, Tokyo, Japan).

## Results

### Comparison of gene expression profile before treatment and response of treatment

First, the gene expression profile before treatment was compared among patients in Cohort 1 with good response (persistent remission for 18 months, n = 9) versus with poor response (relapse after remission or no remission, n = 3). No gene showed a significant difference in expression among the patients; thereby, no correlation was indicated between the gene expression profile before treatment and the response.

### Alteration of gene expression profile by treatment

**Figure 1:**
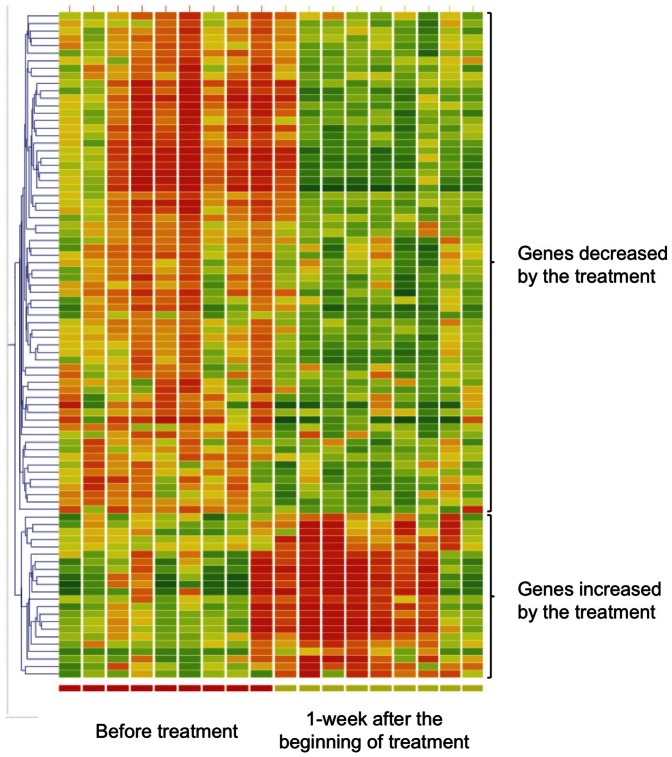
Gene chip analysis of peripheral blood samples obtained from MPA patients (n = 9) with good response (persistent remission) in Cohort 1. The normalized signal values of each gene were compared between before and 1-week after the beginning of treatment. First, genes that showed more than 1.5-fold change in expression level between before and 1-week after the beginning of treatment were extracted. Next, genes that exhibited a significant difference between before and 1-week after the beginning of treatment (p<0.05 in Student's *t*-test assisted by the Benjamin and Hochberg False Discover Rate (FDR) of 0.05) were distilled. As a result, 88 genes were nominated as indicators that reflected a characteristic alteration in expression by the treatment (fold change>1.5, p<0.05 in Student's *t*-test with FDR of 0.05). Hierarchical clustering analyses (Similarity measure: Person correlation, Clustering algorithm: Average linkage) were performed concerning the 88 genes and 18 samples from 9 cases.

When the gene expression profile obtained from 9 patients with good response in Cohort 1 was compared between before and 1-week after the beginning of treatment, 88 genes showed a statistically significant alteration (Figure 1). Among the 88 genes, the expression of 66 genes was significantly decreased, while expression of the other 22 genes was significantly increased by the treatment. On the other hand, no gene in the peripheral blood showed a significant alteration when the samples from 3 patients with poor response in Cohort 1 were examined (data not shown). Although the sample number was limited, these findings suggest the possibility that the response to the treatment may be predicted based on the characteristic alteration of gene profile in the peripheral blood at an early point during the therapy.

### Identification of most valuable genes to predict response to treatment

**Figure 2:**
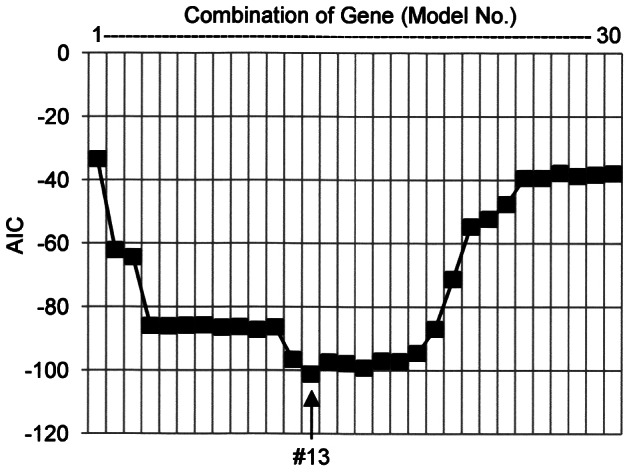
Identification of the most valuable genes to predict the response to the treatment in patients with MPA. Among 88 genes that showed a significant alteration in expression by the treatment, 30 statistically reliable genes were selected for further investigation using TaqMan Low Density Array. Discrimination analysis was conducted concerning 22 patients in Cohort 2, including 17 patients with good response (persistent remission) and 5 patients with poor response (relapse after remission or no remission) as described in the section of Materials and Methods. During the procedure, 30 combinations of genes (model #1 - model #30) were examined for prediction of the response to the treatment. In order to identify the most adequate model among the 30 models, AIC was applied. The model that exhibited the minimum AIC value (model #13) was regarded as the most adequate model, including the most valuable genes to predict the response to the treatment.

In order to identify the most valuable genes to predict the response to the treatment, 30 statistically reliable genes were selected from the 88 genes. The list of the 30 genes is shown in Table 3. These 30 genes included 26 genes significantly decreased and 4 genes significantly increased by the treatment. Next, peripheral blood samples obtained from 22 patients in Cohort 2 were subjected to discrimination analysis using the low density array. Data mining indicated that model #13 showed the minimum AIC value, which meant that the model contained the most valuable genes to predict the response to the treatment (Figure 2). The model #13 contained 13 genes decreased and 3 genes increased by the treatment. The 13 genes are as follows: interferon (IFN) regulatory factor 7 (IRF7); IFN-induced protein with tetratricopeptide repeats 1 (IFIT1); IFIT5, 2′-5′-oligoadenylate synthetase-like (OASL); Charcot-Leyden crystal protein (CLC); guanylate binding protein 1 (GBP-1); proteasome (prosome, macropain) subunit, beta type, 9 (PSMB9); hect domain and RLD 5 (HERC5); chemokine (C-C motif) receptor 1 (CCR1); CD36; membrane-spanning 4-domains, subfamily A, member 4 (MS4A4A); XIAP-associated factor-1 (BIRC4BP); and phospholipid scramblase 1 (PLSCR1). The 3 genes increased by the treatment included defensin α1 and α3 (DEFA1 and DEFA3), defensin α4 (DEFA4), and collagen type IX α2 (COL9A2).

Moreover, in the logistic analysis concerning the genes listed in Table 3, PLSCR1, one of the genes nominated by the discrimination analysis, was identified as the best predictor of the response to the treatment in MPA patients.

## Discussion

This pilot study discovered, for the first time, a set of genes that potentially indicates the response to the treatment in patients with MPA at an early point during the therapy. The gene set consisted of 16 genes present in peripheral blood, including IRF7, IFIT1, IFIT5, OASL, CLC, GBP-1, PSMB9, HERC5, CCR1, CD36, MS4A4A, BIRC4BP, PLSCR1, DEFA1/DEFA3, DEFA4, and COL9A2.

Over the past decade, transcriptome analysis has been energetically performed to identify both diagnostic and prognostic biomarkers of diseases. This has been very successful in the field of oncology, in which gene expression signatures of neoplasm are well associated with the biological behavior and response to treatment. Transcriptome analysis also provides insight into the underlying molecular pathology of the neoplasm [13]. In immune-mediated diseases, peripheral blood samples, e.g., total leukocytes and peripheral blood mononuclear cells (PBMCs), instead of biopsy specimens from the neoplasm have been used for examining gene expression profile [14]. However, unlike oncology, results are sometimes elusive. Since there are plural subsets of cells in peripheral blood, the gene signatures, if any, might be compensated when total leukocytes or PBMCs are subjected to the analysis. In addition, the timing of blood sampling might influence the results. Recently, Lyons and colleagues reported that transcriptome analysis of leukocyte subsets, but not PBMCs, enabled the identification of gene signatures of AAV [15]. Similarly, Mckinney *et al.* reported that the CD8^+^ T cell transcription signature could predict prognosis in autoimmune diseases, including AAV [16]. In the present study, although no correlation was determined between the gene profile of peripheral blood obtained from patients with MPA before treatment and the outcome of the treatment, a characteristic alteration of the gene profile by the treatment at an early point during the therapy was revealed. It seemed likely that the addition of an external factor, that is treatment, made the gene signatures clearer, and that the timing of blood sampling (1-week after the treatment) was appropriate for detection of the signatures.

The 16 genes identified in the present study included 13 genes significantly decreased and 3 genes significantly increased by the treatment. The interaction between the genes that showed significant alteration of expression by the treatment and the pathogenesis of MPA should be considered. It should also be considered whether the alteration of gene expression reflected the effects of the treatment. The 13 genes significantly decreased by the treatment included some IFN-related genes, such as IRF family genes, IFIT family genes, and OAS family genes. These genes are closely related to type 1 IFN and are critically implicated in the pathogenesis of systemic lupus erythematosus (SLE) [17]–[19]. However, it has been reported that the IFN signature of CD4^+^ T cells is not apparent in MPA patients compared with SLE patients [15]. Therefore, the decreased expression of IFN-related molecules, such as IRF7, IFIT1, IFIT5, and OASL, in the present study is unlikely to reflect the pathogenesis of MPA. These IFN-related molecules are mainly expressed in monocytes. Although the number of monocytes was not measured in this study, the reduction of monocytes could be achieved by the treatment. Similarly, the reduction of the gene that codes for CLC protein was regarded as a result of the treatment. CLC proteins are mainly expressed in eosinophils, and the number of eosinophils is rapidly reduced by corticosteroid treatment. These issues should be confirmed in future studies by examining whether the numbers of monocytes and eosinophils in peripheral blood would be actually decreased by the treatment. However, the adequate reduction of the IFN-related molecules and CLC proteins in peripheral blood could be critical for good response to the treatment. The decrease in expression of proinflammatory genes, such as IFN-related molecules and CLC, after starting the therapy in MPA patients with good response may simply indicate the individual strength of anti-inflammatory response to the treatment employed according to the protocols. In other words, stronger immunosuppressants might be needed for patients with poor response.

The genes increased by the treatment included defensins. The induction of defensin genes is also interpreted as the effect of the treatment because circulating neutrophils that express defensins are transiently increased by corticosteroids. Interestingly, the mRNA expression of defensins was up-regulated in peripheral blood in SLE patients, but it was reduced by corticosteroid therapy [20], [21]. Therefore, the contradictory response of the defensin genes to corticosteroid therapy between SLE patients and MPA patients possibly suggests the difference in the pathogenesis of these diseases.

The discrimination analysis nominated 16 genes as distinguished indicators, of which alteration of expression at an early point during the therapy was related to the response to the treatment. Since the number of genes (16) is too much for routine clinical analysis, further efforts to extract the best predictors among them are needed. As a trial for this purpose, logistic analysis was conducted independently to the discrimination analysis. The result indicated PLSCR1, one of the genes nominated by the discrimination analysis, as the sole statistically significant predictor at this time. This result, namely the extraction of a single gene, might be related to the limitation of this study with small sample size. We expect that further investigations with more samples would extract the best predictors among the 16 genes, including PLSCR1.

It remains elusive whether differences in treatment, including oral corticosteroids, intravenous methylprednisolone, immunosuppressive agents, and plasmapheresis, have an effect on the results of gene expression in this study. Further sub-analysis is not feasible due to the limited number of patients. However, it is considered that sufficient alteration of the nominated genes suggests the appropriate strength of the treatment. Thus, the relative intensity of treatment against the disease activity, but not the modality of treatment itself, could affect the results of gene expression in this study.

The remaining challenge is the validation of the results. In order to validate the practical significance of the prediction using the 16 genes nominated in this study, as well as to establish a guideline for the management of patients with MPA, larger clinical trials should be conducted.

## Supporting Information

Table S1 **Comparison of clinical characteristics between male and female genders in Cohort 1 and 2.**
(DOC)
