# Supplementary material for: Prediction of Response to Treatment by Gene Expression Profiling of Peripheral Blood in Patients with Microscopic Polyangiitis
Source: PLoS One. 2013 May 17;8(5):e63182. doi: 10.1371/journal.pone.0063182 (PMC3656865; doi:10.1371/journal.pone.0063182)
Supplement: Table S1 — Comparison of clinical characteristics between male and female genders in Cohort 1 and 2. (DOC) [file pone.0063182.s001.doc]

Table S1. Comparison of clinical characteristics between male and female genders in Cohort 1 and 2

| Clinical characteristics | Male (n=11) | Female (n=22) | p-value* |
| --- | --- | --- | --- |
| Age | 67.9 ± 5.9 | 65.2 ± 11.9 | 0.8482 |
| Serum creatinine (mg/dL) | 2.07 ± 1.20 | 1.70 ± 1.68 | 0.1219 |
| Serum MPO-ANCA (U/mL) | 353.5 ± 197.1 | 501.4 ± 1060.7 | 0.2764 |

*Mann-Whitney *U*-test.
